# Editorial: The rocky biosphere: New insights from microbiomes at rock-water interfaces and their interactions with minerals

**DOI:** 10.3389/fmicb.2022.1102710

**Published:** 2022-12-08

**Authors:** Yohey Suzuki, Elizabeth Trembath-Reichert, Henrik Drake

**Affiliations:** ^1^Department of Earth and Planetary Science, The University of Tokyo, Tokyo, Japan; ^2^School of Earth and Space Exploration, Arizona State University, Tempe, AZ, United States; ^3^Department of Biology and Environmental Science, Linnaeus University, Kalmar, Sweden

**Keywords:** geobiology, geomicrobiology, astrobiology, subsurface microbiology, deep biosphere

The deep subsurface hosts one of the largest, yet least explored ecosystems on Earth. Exploration of the deep biosphere has implications for our understanding of the sustainability of our planet, particularly in ecosystems where life faces the extreme deprivation of energy and nutrients (Hoehler and Jørgensen, 2013). Our knowledge of lifeforms and processes in this ecosystem also have far-reaching astrobiological implications that influence exploration strategy of planetary bodies such as Mars and icy moons (Onstott et al., 2019). Microbial communities in the subsurface account for a significant proportion of global biomass on Earth (McMahon and Parnell, 2014; Bar-On et al., 2018; Magnabosco et al., 2018; Wang et al., 2021). These subterranean communities may also help us to understand the evolutionary history of microbial life on our planet. A recent study demonstrated the slow evolution of subsurface microbes (Becraft et al., 2021), thus primitive characters might be retained. Additionally, biosignatures of deep ancient life might be omnipresent as fossilized biofilms and isotope fingerprints in deep fracture coatings (Ivarsson et al., 2020). Research studies on live communities in the deep rock-hosted biosphere are restricted by the hard-to-reach nature of this ecosystem. Access to the deep biosphere requires deep sea drilling, deep continental drilling, or relying on existing subsurface infrastructure (tunnels, mines, research facilities). These limitations call for general conceptual studies, as well as detailed site-specific studies for various settings and physico-chemical conditions. In this Research Topic, studies from deep continental settings and oceanic settings are presented and describe both fluid-bound communities and biofilm communities, as well as a review that summarizes the state of the field.

Chemoautotrophic archaea are globally distributed in deep-sea hydrothermal vent and sub-vent ecosystems. At the Mid-Cayman Rise, hydrogenotrophic methanogens from the genus *Methanothermococcus* comprised the most abundant lineage of archaea observed in venting fluids, yet how they adapt and diversify in these habitats remain largely unknown. To determine genomic variation and selection pressure within methanogenic populations at vents, Hoffert et al. examined five *Methanothermococcus* single cell amplified genomes (SAGs) as well as 15 metagenomes and 10 metatranscriptomes. Their results show that presence of prophage sequences and accessory functions such nitrogen fixation and the CRISPR/Cas immune response does not confer selective advantages. In addition, the accumulation rate of single nucleotide variants was high in *Methanothermococcus* genomes, suggesting that *Methanothermococcus* lineages are maintained in high abundances at the vent sites. This work highlights the power of combining single-cell, metagenomic, and metatranscriptomic datasets to determine how evolution shapes microbial abundance and diversity in hydrothermal vent ecosystems.

Fluid circulation through oceanic crust plays important roles in sustaining microbial habitats and global biogeochemical cycling. However, studying rocky habitats is challenged by sampling logistics and low biomass. By using small volumes of low biomass (approximately 103 cells ml^−1^) crustal fluid from borehole observatories installed at the North Pond study site on the western flank of the Mid-Atlantic Ridge, D'Angelo et al. applied redox-sensitive fluorescent molecules to flow cytometric sorting of cells for subsequent single cell genomic sequencing. Comparison of data from single cell genomics with previously profiled metagenomic and metatranscriptomic data shows that even with low coverage genome sequencing, sorting cells from < 1 ml of crustal fluid results in similar taxonomic and metabolic profiles as conventional omics approaches that require orders of magnitude higher fluid volumes. Their results reconfirmed that the diverse community dominated by Gammaproteobacteria, Bacteroidetes, Desulfobacterota, Alphaproteobacteria, and Zetaproteobacteria. Gammaproteobacterial members had genes for the fixation of carbon and nitrogen, whereas those of Bacteroidetes were annotated as putative heterotrophs. These results strengthen the technical advantage of fluorescence activated cell sorting as an alternate molecular tool for low biomass ecosystems ubiquitously encountered in the rocky biosphere.

In the deep terrestrial biosphere, Nuppunen-Puputti et al. enriched subsurface microbial communities on mica schist in microcosms containing bedrock groundwater from the depth of 500 m from Outokumpu, Finland. The biofilms revealed numerous different microbial cell morphologies and attachment strategies on the schist surface. Bacteria with outer membrane vesicle-like structures, hair-like extracellular extensions, and long tubular cell structures expanding over hundreds of micrometers were observed. Amplicon sequencing revealed domination of *Pseudomonas, Desulfosporosinus, Hydrogenophaga*, and *Brevundimonas* after up to 40 months of incubation. Indication for a high degree of environmental adaptivity to oligotrophic environment and potential for shifting between multiple energy or carbon sources were derived from the metagenome assembled genomes (MAGs) from communities involved in the biofilm formation. The MAGs also suggest ubiquitous organic carbon oxidation and capacity for arsenate and selenate reduction. The authors propose that the observed interaction between the deep subsurface microbial communities and the rock surfaces might be crucial for sustaining life under oligotrophic conditions in the deep subsurface environment of crystalline bedrock.

The deep subsurface hosts the significant portion of prokaryotic biomass on modern Earth, which might be analogous on early Earth and modern Mars where the surface environment is harsh even for microbial life. The rocky biosphere is not sustained by photosynthetic energy sources but by those derived from rock-water interactions. Takamiya et al. provide a mini-review describing the current understanding of the rocky biosphere based on inorganic and organic energy sources independently from photosynthesis. Such energy sources are supplied in the deep subsurface where water radiolysis occurs at uranium ore deposits, while serpentinization produces H_2_ and hydrocarbons. Advances in omics-based approaches and nanosolid characterizations have unveiled taxonomic and metabolic features of microbiomes deeply hosted in the oceanic and continental crusts in association with mineral assemblages prevalent on early Earth and other planetary bodies potentially harboring extant life.

Olivine is widely available in ocean crust on Earth, as well as other astrobiology targets, and can provide an energy source for chemolithoautotrophs *via* serpentinization reactions. Smith et al. provide the metagenome assembled genome (MAG) of a novel acetogen, *Candidatus* Acetocimmeria pyornia, from an olivine biofilm in the basaltic crust of the Juan de Fuca Ridge (JdFR). The samples were retrieved from 4-year incubations of rocky substrates within the crustal fluids of the JdFR borehole observatory. This MAG may represent a new lineage of acetogens within the class Clostridia. The MAG encodes the complete Wood-Ljungdahl pathway, a metabolically flexible and potentially ancient carbon metabolism, as well as genes for use of molecular hydrogen and import of metallic cations that could further support an organism adapted to life in basalt crust biofilms. This study furthers our knowledge of deep subsurface microbial communities that shape ocean geochemistry.

While the deep subsurface is known to contain a vast reservoir of microbial life, the spatial heterogeneity of microbes and their potential mineral substrates complicate our ability to constrain bulk estimates of abundance and activity. Casar et al. have addressed this issue by interrogating the relationship between biofilm biomass and host rock minerology via *in situ* cultivation of biofilms on native rocks with coupled microanalysis techniques. Their results from the Deep Mine Microbial Observatory in South Dakota suggest mineral selectivity plays a role in biofilm formation, which is a key constraint on subsurface systems considering biofilms are estimated to account for most of the biomass in the subsurface. The observed mineral preferences have additional implications for understanding bulk cycles of iron and sulfur bearing minerals in deep continental settings.

Microbial communities sequestered in subsurface fluids can be separated from surface interactions for hundreds to millions of years. Sheik et al. interrogated how potential nutrient limitation(s) in such sequestered fluids might structure microbial communities in subsurface brines from the Soudan Underground Mine in Minnesota. From metagenome assembled genomes (MAGs) they recovered evidence for carbon, nitrogen, sulfur, and hydrogen elemental cycling and provide further examples of potential deeply branching lineages from subsurface systems. They also provide additional evidence that the byproducts of the osmolyte glycine-betaine may fuel methylotrophy in subsurface systems as no other forms of methanogenesis were recovered from MAG analyses. Overall, these findings support theories that metabolically versatility may be a key trait for subsurface survival.

The rocky-biosphere is one of the least understood biospheres. With this Research Topic, we have gathered a collection of timely additions to take the knowledge of this realm further, both in the oceanic and continental deep biospheres. The interpretations presented in this Research Topic are dominantly based on metagenome assembled genomes, metatranscriptomes and amplicon sequencing of indigenous communities from oligotrophic environment, as well as from long-term incubations of these type of communities. The results of the studies presented here increase our knowledge of deep subsurface microbial communities, including their community structure, biofilm formation and metabolic versatility.

The oceanic and continental rocky subsurface biosphere is one of the least understood microbial ecosystems. The studies of this Research Topic expand our knowledge of community structure, biofilm formation and metabolic versatility of this realm, drawing on MAGs, metatranscriptomes and amplicon sequencing of indigenous communities from oligotrophic environments, as well as long-term incubations of these communities. Our future efforts need to be directed to understand the correlation between environmental variables and phylogenomic properties of microbial communities and to constrain the limit of the deep rocky biosphere with respect to temperature, depth, and nutrient availability.

## Author contributions

All authors listed have made a substantial, direct, and intellectual contribution to the work and approved it for publication.
